# Zeolite Blending: A New Approach to Direct Crystallization of Aluminosilicate Zeolite

**DOI:** 10.1002/anie.202424442

**Published:** 2025-02-19

**Authors:** Masato Sawada, Kensuke Okubo, Yao Lu, Samya Bekhti, Hiroto Toyoda, Liang Zhao, Hiroaki Onozuka, Susumu Tsutsuminai, Junko N. Kondo, Hermann Gies, Toshiyuki Yokoi

**Affiliations:** ^1^ Institute of Integrated Research Institute of Science Tokyo 4259 Nagatsuta Midori-ku Yokohama Japan; ^2^ Science & Innovation Center Mitsubishi Chemical Corporation 1000 Kamoshida-cho Aoba-ku Yokohama Japan; ^3^ Office of Communication and DEI Section Institute of Science Tokyo 4259 Nagatsuta Midori-ku Yokohama Japan; ^4^ Institute of Geology, Mineralogy und Geophysics Ruhr-University Bochum 44780 Bochum Germany; ^5^ World Research Hub (WRH) Institute of Science Tokyo 4259 Nagatsuta Midori-ku Yokohama Japan

**Keywords:** Zeolite, Interzeolite conversion/transformation, CON-type topology, Building-units

## Abstract

Various synthesis methods for zeolites have been developed to date; however, precise control over aluminum (Al) content remains a significant challenge. For instance, in the case of CON‐type zeolites, it is especially difficult to obtain a Si/Al ratio below a value of approximately 100 by direct synthesis, and below approximately 30 by post‐treatment. In this study, we have developed a novel direct synthesis method of the CON‐type zeolite having desired Al content by applying the interzeolite conversion/interzeolite transformation, wherein precursor zeolites that possess common composite building units (CBUs) with the target zeolite are employed as the Al and Si source. Initially, a single zeolite was utilized as a precursor, resulting in the successful synthesis of a high Al CON‐type zeolite (Si/Al=40). Finally, we developed a novel synthesis method of the CON‐type zeolite; blending of two types of zeolites, MFI and Beta, as starting material in the IZC method led to the direct crystallization of the CON‐type aluminosilicate with high Al content (Si/Al=20).

## Graphical abstract

The blending of two types of zeolites, MFI and Beta, as raw material in the interzeolite conversion/transformation method resulted in the collection of the required composite building‐units of the CON‐type topology, leading to the direct crystallization of the CON‐type aluminosilicate with high Al content (Si/Al=20).

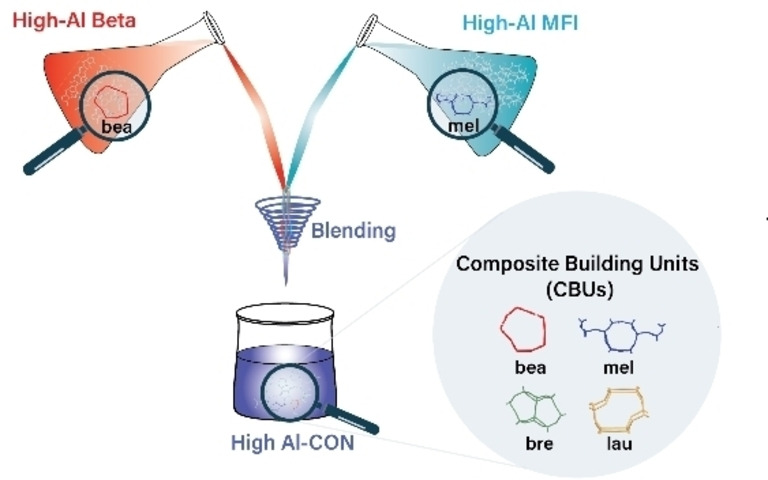

Zeolites are crystalline porous aluminosilicates with ordered channels and cavities.[^1^, ^2^] Due to the acidic OH groups that bridge to aluminium and silicon atoms in aluminosilicate framework, they are widely used in industry as solid acid catalysts.^[2]^ Numerous efforts including bottom‐up and top‐down approaches, e.g., the direct crystallization of amorphous starting materials and the post‐modification of zeolite, have been dedicated to synthesize zeolites with a wide range of elemental compositions, particularly Al contents. Besides, the interzeolite conversion/transformation method has been developed and attracted much attention. This is based on the structural conversion of zeolite through the dissolution followed by re‐crystallization, and has widely been employed in synthetic studies due to its capability to form structures that are not achievable through conventional crystallization techniques.^[3]^ Moreover, it enables the production of zeolites with high crystallinity.[^4^, ^5^, ^6^] Currently, this method relies on the use of single zeolite as starting material, and its selection is based on the common composite building‐units (CBUs) of parent and targeted zeolites; CBUs are clusters of TO_4_ units where the T atoms consist of either Si, Al, or B, to be of significant importance in determining the structure of the final products. However, the use of more than two types of zeolites as starting material has never been studied so far.

CON‐type aluminosilicate zeolites have been regarded as a promising catalyst for the selective production of light olefins in the methanol to olefin (MTO) reaction due to their distinctive structure;^[7]^ the 10‐ and 12‐ring (10R and 12R) channels are interconnected to form large voids at the intersections (Figure S1). After this discovery, the extensive studies on the CON‐type zeolite have been conducted. We have studied on the crystallization process of the CON‐type zeolite.^[8]^ Very unique “ultrafast flow synthesis of CON‐type zeolite has been developed.^[9]^ Creation of mesopores inside the crystal have successfully been achieved by post‐ and direct method, improving the MTO performance.[^10^, ^11^] The bead‐milling, recrystallisation and defect‐healing treatments have been adapted to yield highly crystalline zeolite nanoparticles, showing prolonged lifetime in the MTO reactions.^[12]^ Furthermore, CIT‐1 exhibits superior hydrothermal stability relative to ZSM‐5,^[13]^ and its distinctive structure comprising both large and middle pores offers considerable potential for applications in other catalytic reactions. Thus, CON‐type aluminosilicate zeolite is a really promising solid‐acid catalyst, while one of the drawbacks is the narrow compositional variations of the framework; the direct synthesis process results in the production of the CON‐type zeolite with a relatively low aluminum content and a restricted Si/Al ratio, ranging from 100 to infinity within the framework.[^7^, ^11^] Although the post‐modification represents a viable method to increase the Al content, there are concerns on the content, state and distribution of the Al atoms. On the other hand, computation research demonstrated that the CON‐type aluminosilicate with Si/Al of 6 is ideally possible, and T2 site among 7 T sites in the CON topology is found to be energetically favorable.^[14]^ If we could extend the Al content in this zeolite, diverse applications will be developed. Therefore, the development of a novel synthesis method for the CON‐type zeolites with a wide range of elemental compositions has strongly been desired.

In this study, the interzeolite conversion/transformation (IZC) method was employed for the synthesis of high Al‐containing CON‐type zeolite. Finally, we have succeeded in the development of novel synthesis method of the CON‐type zeolite; blending of two types of zeolites, MFI and Beta, as starting material in the IZC method led to the direct crystallization of the CON‐type aluminosilicate with high Al content.

First, we tried to synthesize high Al‐containing CON‐type zeolite via the IZC method using single starting material. Beta zeolite and MFI‐type zeolite, which contain common CBU with CON‐type zeolite (Figure S2) were selected as stating zeolite. In order to ascertain the significance of the common CBU, FAU‐type zeolite (Si/Al=15), which did not contain any common CBUs, was used as a control. In addition, amorphous silica was used to adjust the Si/Al ratio. CON‐type zeolites were hydrothermally synthesized according to the previously reported method.[^7^, ^11^] The CON‐type zeolites obtained from Beta, MFI‐type and FAU‐type zeolites are abbreviated as CON‐Beta‐*X*, CON‐MFI‐*X*, and CON‐FAU‐*X*, respectively, where *X* means the Si/Al ratio in the mother gel.

As observed from the XRD patterns (Figure 1), the crystallization of CON‐type zeolite did not proceed when FAU‐type zeolite was used as the precursor (Figure 1 (d)). However, the use of Beta or MFI‐type zeolite, which possess a common CBU with the target zeolite, as the precursor resulted in the successful synthesis of CON‐type zeolite. (Figure 1 (b) and (c)). It was thus demonstrated that the use of zeolite precursors containing CBUs common to the target zeolite enabled the synthesis of high Al containing CIT‐1. The CON‐Beta‐40 was obtained in an almost pure phase (yield is 94 %, Si/Al ratio is 41), whereas the CON‐MFI‐40 still contained MFI mixed in (yield is 85 %, Si/Al ratio is 38). It is therefore concluded that beta zeolites represent the optimal precursor zeolite for the enhancement of CON‐type zeolite crystallization, with the expectation of an Al content close to or below Si/Al=40. One of the advantages for IZC method is the decrease in the crystallization period. Generally, the CON zeolite requires a long crystallization period; for example, CON‐400 was crystalized after the hydrothermal treatment at 170 °C for 7 days. Note that, we found that the CON‐Beta‐40 was fully crystalized within 2 days (Figure S3).

**Figure 1:**
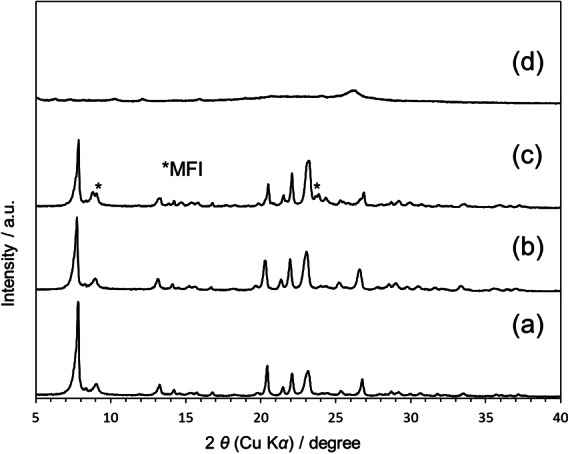
XRD patterns of CON‐type zeolites; (a) CON‐400, (b) CON‐Beta‐40, (c) CON‐MFI‐40, and (d) CON‐FAU‐40. (a) was the conventional CON‐type zeolite with Si/Al ratio of 400.

The obtained samples were also compared with the conventional CON‐type zeolite with Si/Al ratio of 400 (CON‐400). The SEM images of both CON‐400 and CON‐Beta‐40 (Figure S4 (a) and (b)) showed no apparent differences in particle morphology. Furthermore, the ^27^Al MAS MNR spectra of the products (Figure S5), exhibit a peak at 50–60 ppm which is attributed to tetrahedrally coordinated Al species in the framework. Additionally, small peaks at around −0.5 ppm are associated with octahedrally coordinated Al species. The ^27^Al MAS NMR results confirmed that the Al atoms are well introduced into the framework.

As shown in Table 1, the Si/Al molar ratio of CON‐Beta‐40 was approximately 40. indicating that the Al composition in the initial reactant gel and the resulting zeolite are identical. This suggests that the product is balanced, additionally, the acid amount was significantly high due to the high Al content in the starting material. The acid strength of CON‐Beta‐40 was confirmed by comparison with CON‐400 through the use of NH_3_‐TPD (Figure S6). Although the peak top temperature may vary due to differences in particle size and other conditions, which makes it difficult to draw definitive conclusions, it is possible that CON‐Beta‐40 exhibits stronger acid strength. CON‐MFI‐40, which contains larger secondary particles than CON‐400 and CON‐Beta‐40 (Figure S4 (c)), exhibited a smaller acid amount than CON‐Beta‐40 (Table 1). There is a significant difference in the state of tetrahedrally coordinated Al species between CON‐Beta‐40 and CON‐MFI‐40 (Figure S5). It is known that CON‐MFI‐40 contains a significant amount of 6‐coordinate Al, which may have contributed to the lower acid content observed in this sample. The broader NMR peak of tetrahedrally coordinated Al species in CON‐MFI‐40 compared to CON‐Beta‐40 (Figure S5) suggests significant variations in Al distribution across different T‐sites, with a marked increase in the proportion of Al atoms located around 53.6 ppm. We assign this 53.6 ppm peak to Al atoms residing in the *mel* units of the CON zeolite. This indicates that Al atoms in CON‐MFI‐40 predominantly occupy *mel* units (derived from the MFI precursor), while those in CON‐Beta‐40 are more evenly distributed within the *bea* and other structural units. The broader *h* peak in the NH_3_‐TPD profile of CON‐MFI‐40 (Figure S6) further reflects these structural differences, as the Al sites in *mel* units would result in stronger acid sites and higher desorption temperatures. For the synthesis mechanism, it is concluded that the target zeolite framework was formed from locally ordered aluminosilicate species, which originated from the decomposition of starting materials that are structurally similar to the target zeolite. Concretely, the crystallization of CON‐type zeolites proceeded from Beta or MFI‐type zeolite having common CBU to the target zeolite.[^15^, ^16^, ^17^, ^18^] By this method, the CON‐type zeolites with Si/Al ratio of 40 was successfully synthesized in a pure‐phase. The Si/Al molar ratio of CON‐Beta‐40 and CON‐MFI‐40 was approximately 40, this strategy enabled the synthesis of zeolite framework beyond the present limit of Si/Al ratio region in direct synthesis. In addition, because Al atoms are well introduced in the framework, the synthesized CON‐type zeolites would be applied for reactions requiring relatively high acid strength.

**Table 1:** Physicochemical properties of the CON‐type zeolites.

CON‐	Si /Al^[a]^	Si /B^[a]^	A_acid_^[b]^ /mmol (g‐cat)^−1^	S_BET_^[c]^ /m^2^g^−1^	V_micro_^[c]^ /cm^3^g^−1^	S_ext_^[c]^ /m^2^g^−1^
400	431	31	0.018	633	0.24	43
Beta‐40	41	51	0.270	670	0.24	46
MFI‐40	38	50	0.166	515	0.23	24

^[a]^ ICP‐AES, ^[b]^ NH_3_‐TPD, and ^[c]^ N_2_ adsorption‐desorption measurement

More detailed properties of the high Al‐containing CON‐Beta‐40 were investigated in comparison with the sample prepared by the post‐treatment (CON−P‐30, Si/Al=30). CON−P‐30 was prepared through the deboronation and Al‐introduction to the borosilicate (Si/B=300).^[19]^ XRD patterns of CON‐Beta‐40 and CON−P‐30 were almost identical (Figure S7), while the differences in the particle morphology, local structure of framework Al and acidic/catalytic properties were found. First, the particle size of CON‐Beta‐40 was smaller than that of CON−P‐30 (Figure S8) resulting in the larger external surface area (Table S1). This is due to the larger particle size of the original B‐CON in comparison with Al‐CON.[^7^, ^19^, ^20^] Second, the peaks attributed to tetrahedrally coordinated Al atoms in ^27^Al MAS NMR spectrum of CON‐Beta‐40 were slightly shifted to higher field compared to those of CON−P‐30 (Figure S9). It has been well‐recognized that the chemical shift of tetrahedrally coordinated Al atoms is decreased with an increase in the average TOT angle.[^21^, ^22^] Based on this finding, more Al atoms in CON−P‐30 are located at narrow TOT angle sites, while, those in CON‐Beta‐40 would preferentially be located at the wide TOT angle sites, i.e., relatively larger, more spacious environments. As reported in studies on the Al position in MFI‐type zeolites during MTO reactions,[^23^, ^24^] Al sites located within the channels are less mobile and preferentially generate higher olefins, which can transform into aromatics and carbon precursors that are difficult to diffuse out. Given its wider pore structure and higher Al content, CON‐Beta‐40 is expected to exhibit less deactivation in the MTO reaction compared to CON−P‐30. Al species in CON‐Beta‐40 was confirmed to the cross sections “a,” and “b” in Figure S10 whereas CON−P‐30 has the cross sections only “a” in the ^27^Al MQMAS NMR spectra. This trend was similar to the previous report, concluding that framework Al atoms corresponding to the cross section “b” contribute to the suppression of the formations of aromatics and heavy hydrocarbons in MTO reaction.[^7^, ^25^, ^26^] Lastly, the acid density on the external surface of CON‐beta‐40 was lower than that of CON−P‐30 as shown below.

In order to estimate the reactivity of Brønsted‐acid sites on the external surface, the cracking reaction of 1,3,5‐trisopropylbenzene (TIPB) was conducted(Table 2)^[27]^ . The initial conversion of TIPB over both samples was nearly identical, while the external surface area of CON‐Beta‐40 was considerably larger than that of CON−P‐30. Therefore, the acid density of CON‐Beta‐40 is lower than that of CON−P‐30. It is well established that acid sites on the external surface of z eolites form coke on the outer surface, which leads to deactivation.^[28]^ Therefore, it can be hypothesized that CON‐Beta‐40 will exhibit superior catalytic lifetime than CON−P‐30 in the MTO reaction. The aforementioned characteristics of CON‐Beta‐40 prompted us to investigate its catalytic properties.

**Table 2:** 1,3,5‐triisopropylbenzene (TIPB) conversion reaction over direct and post synthesis CON‐type zeolites.

Sample	Initial Conv.	External surface area^[a]^/m^2^g^−1^
CON‐Beta‐40	78.2 %	47
CON−P‐30	76.5 %	29

^[a]^ N_2_ adsorption‐desorption measurement. Reaction conditions; 20 mg catalyst, 10 μL/min TIPB diluted with Ar (10 mL min^−1^), W/F=21 g h mol^−1^, and reaction temp.: 400 °C.

Here, the catalytic property of CON‐Beta‐40 was studied by *n*‐hexane cracking as a model reaction over strong acid sites.[^29^, ^30^, ^31^, ^32^] As a control, MFI‐40 (ZSM‐5, Si/Al=40) and Beta‐50 (Beta zeolite, Si/Al=50) were tested (Figure 2). As we expected, MFI‐40 exhibited a stable and high catalytic performance compared to Beta‐50. Note that CON‐Beta‐40 exhibited an intermediate performance between MFI‐40 and Beta‐50. The P/E (propylene/ethylene) ratios at the initial activity for MFI‐40, CON‐Beta‐40 and Beta‐50 were calculated to be 2.4, 3.9 and 5.1, respectively. This difference would be caused by the differences in the topology as well as acidic properties.[^33^, ^34^] These results indicate that CON‐type zeolites with high Al content, may be suitable for reactions that require a relatively high acid strength.The high catalytic activity of CON‐Beta‐40 in the hexane cracking reaction prompted us to synthesize the CON‐type zeolites with an even higher Al content. The maximum Si/Al molar ratio of Al‐introduced CON‐type zeolites is 30, which was obtained by post‐treatment of directly synthesized borosilicate.[^19^, ^20^] Hence, an attempt was made to embed more Al atoms exceeding the limit for various catalytic reactions.

**Figure 2:**
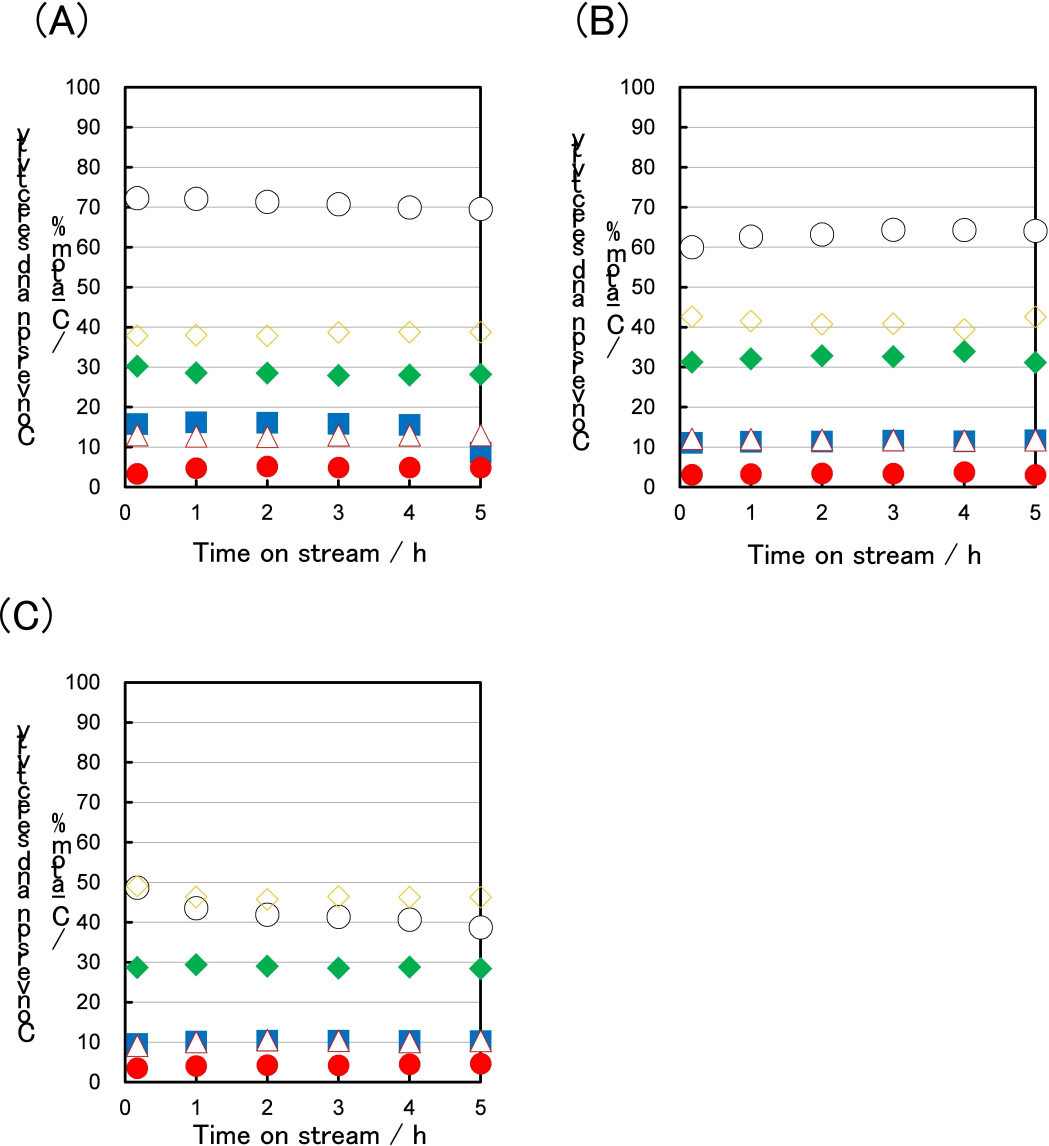
*n*‐hexane cracking reaction; (A) MFI‐40, (B) CON‐Beta‐40 and (C) Beta‐50. Reaction conditions: 20 mg catalyst, 2.1 μL/min n‐hexane diluted with Ar (6.2 mL min^−1^), W/F=20.7 g h mol^−1^, reaction temp.: 600 °C: ○, conversion of *n*‐hexane; ▪, selectivity toward ethene; ◊ , selectivity toward propylene; ▵, selectivity toward butenes; ⧫ , selectivity toward C1−C4 paraffins; •, selectivity toward hydrocarbons (≥ C5).

As indicated in XRD patterns (Figure S11), the peaks derived from the precursor zeolite remained in the case of the synthesis from one type of zeolite (Beta or MFI‐type zeolite) by the IZC method. In an attempt to optimize the synthesis process, we modified the synthesis temperature and crystallization time. Increasing the synthesis temperature to 200 °C resulted in a noticeable decrease in the peak derived from the Beta structure (Figure S12 (b) and (c)). Additionally, extending the reaction time resulted in a further decrease in the peak derived from the beta structure. (Figure S12 (c) and (d)). However, these modifications did not enable the synthesis of pure CON‐type zeolites. We then attempted, blending the two precursor zeolites, namely Beta‐type and MFI‐type, at 170 °C. which led to a slight decrease in the beta structure peak (Figure 3 (b) and (c)).

**Figure 3:**
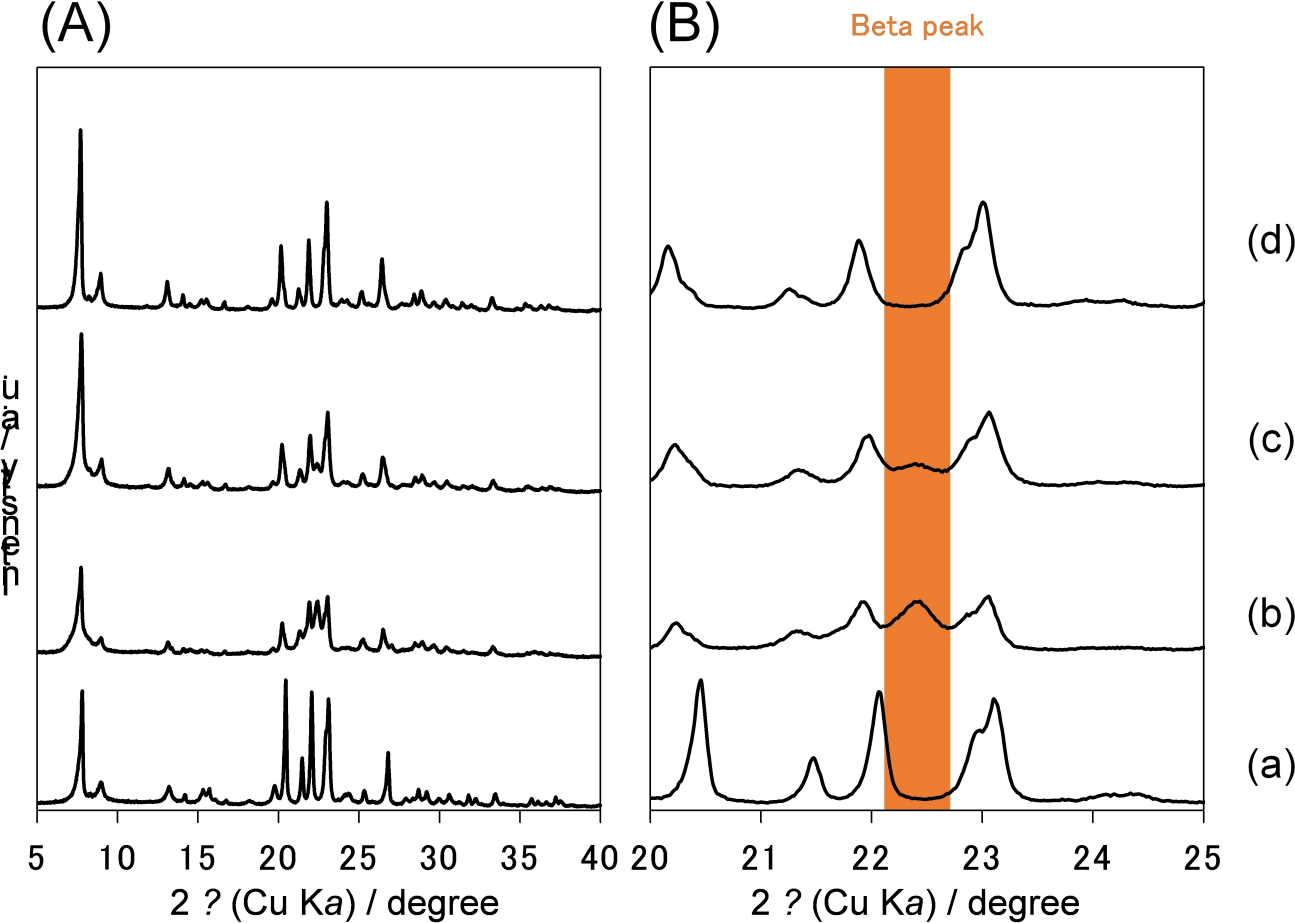
XRD patterns of CON‐type zeolites around the range of (A) 2*θ*=5–40 and (B) 2*θ*=20–25 deg.; (a) CON‐400, (b) CON‐Beta‐20–170 °C −7d, (c) CON‐Beta+MFI‐20–170 °C −7d, and (d) CON‐Beta+MFI‐20–200 °C −7d. (b) was synthesized by using Beta‐zeolite for the synthesis of CON‐type zeolite with Si/Al ratio of 20 at 170 °C 7 days, (c) was synthesized by using Beta and MFI‐type zeolites in a 1 : 1 ratio for the synthesis of CON‐type zeolite with Si/Al ratio of 20 at 170 °C 7 days, (d) was synthesized by using Beta zeolite and MFI‐type zeolite in a 1 : 1 ratio for synthesis CON‐type zeolite with Si/Al ratio of 20 at 200 °C 7 days.

Interestingly, after increasing the synthesis temperature to 200 °C under blending conditions, pure‐phase CON‐type zeolite was successfully synthesized (Figure 3 (d)). This sample was abbreviated as CON‐Beta+MFI‐20. The particle size of CON‐Beta+MFI‐20 in SEM images is larger than that of CON‐400 and CON‐Beta‐40 (Figure S4 (b) and S13 (a) and (b)). Thus, crystallization was evident due to the increase in synthesis temperature (Figure S13 (c)). The acid amount of CON‐Beta+MFI‐20 from NH_3_‐TPD profile was about twice as large as that of CON‐Beta‐40 (Table S2 and Figure S14), which means that it has abundant acid amount for catalytic reactions.

In order to determine the key CBU that dominates the crystallization into the CON‐type zeolite, whether Beta or MFI, we selected extreme ratios in the starting material blend. In the case where 90 wt % of the starting material was MFI‐type zeolite (CON‐Beta: MFI=1 : 9), XRD patterns and SEM images showed that the MFI zeolite remained (Figure S15 (c) and 16 (b)). In the opposite case (CON‐Beta: MFI=9 : 1), CON‐type zeolite was obtained in a single phase without formation of Beta zeolite (Figure S15 (a)). Thus, Beta zeolite was found to be the effective starting material, while a small amount of MFI‐type zeolite assists the synthesis of high Al containing CON‐type zeolite. Therefore, high Al‐containing CON‐type zeolites were synthesized by using precursor zeolites having common CBUs with the target zeolite. In addition, the blending of more than two types of zeolites as starting materials in the IZC method provided a preferential synthetic environment. Further investigations on this belnding method such as the impact of the physicocemical properties of the starting materials and their role are currently under way.

In conclusion, high‐aluminium CON zeolites were successfully synthesized by the IZC method using multiple types of zeolites as starting materials. Initially, a single type of zeolite was employed as the precursor, resulting in the successful synthesis of a CON‐type zeolite with a Si/Al ratio of approximately 40. The resulting sample, CON‐Beta‐40, exhibited a smaller particle size when compared to a zeolite sample obtained through a conventional post‐treatment method. Furthermore, it was confirmed to have a superior Al distribution in the MTO reaction. Subsequently, the synthesis of CON‐type zeolites with an even higher Al content was investigated using a combination of zeolite precursors, a process we designated as the “blending method“. This approach enabled the expansion of the Si/Al ratio range to approximately 20, which could not be achieved with a single zeolite precursor. This strategy represents the first instance of the direct synthesis of zeolites with high Al content on a global scale. Our findings suggest that the required CBUs of targeted zeolite can be collected by blending more than two types of zeolites as starting material. Additionally, given its considerable potential of the newly developed “blending method”, we have high hopes for its further application in the synthesis of zeolites beyond CON‐type zeolites.

## Supporting Information

The authors have cited additional references within the Supporting Information.[^35^, ^36^, ^37^]

## Conflict of Interests

The authors declare no conflict of interest.

## Supporting information

As a service to our authors and readers, this journal provides supporting information supplied by the authors. Such materials are peer reviewed and may be re‐organized for online delivery, but are not copy‐edited or typeset. Technical support issues arising from supporting information (other than missing files) should be addressed to the authors.

Supporting Information

## Data Availability

The data that support the findings of this study are available from the corresponding author upon reasonable request.
