# Supplementary material for: Zeolite Blending: A New Approach to Direct Crystallization of Aluminosilicate Zeolite
Source: Angew Chem Int Ed Engl. 2025 Feb 19;64(11):e202424442. doi: 10.1002/anie.202424442 (PMC11891624; doi:10.1002/anie.202424442)
Supplement: Supplementary file 1 — Supporting Information [file ANIE-64-e202424442-s001.pdf]

Angewandte Chemie
Eine Zeitschrift der Gesellschaft Deutscher Chemiker

Supporting Information

Zeolite Blending: A New Approach to Direct Crystallization of Aluminosilicate Zeolite

*M. Sawada, K. Okubo, Y. Lu, S. Bekhti, H. Toyoda, L. Zhao, H. Onozuka, S. Tsutsuminai, J. N. Kondo, H. Gies, T. Yokoi**

Zeolite Blending: A New Approach to Direct Crystallization of Aluminosilicate Zeolite

Masato Sawada, [a] Kensuke Okubo, [a] Yao Lu, [a] Samya Bekhti, [a] Hiroto Toyoda, [a] Liang Zhao, [a] Hiroaki Onozuka, [b] Susumu Tsutsuminai, [b] Junko N. Kondo, [c] Hermann Gies, [d, e] and Toshiyuki Yokoi*[a, d, e]

[a] M. Sawada, K. Okubo, Y. Lu, S. Bekhti, H. Toyoda, L. Zhao, J. N. Kondo, T. Yokoi
Institute of Integrated Research
Institute of Science Tokyo
4259 Nagatsuta, Midori-ku, Yokohama, Japan
E-mail: yokoi@cat.res.titech.ac.jp

[b] H. Onozuka, S. Tsutsuminai
Science & Innovation Center
Mitsubishi Chemical Corporation
1000 Kamoshida-cho, Aoba-ku, Yokohama, Japan

[c] J. N. Kondo
Office of Communication and DET, DEI Section
Institute of Science Tokyo
4259 Nagatsuta, Midori-ku, Yokohama, Japan

[d] H. Gies
Institute of Geology, Mineralogy und Geophysics
Ruhr-University Bochum
44780 Bochum, Germany

[e] H. Gies, T. Yokoi
World Research Hub (WRH)
Institute of Science Tokyo
4259 Nagatsuta, Midori-ku, Yokohama, Japan

WILEY-VCH

Table of Contents

Experimental Procedures……………………………………………………………………………………………………3

Supporting Results……6

Supplementary References……………………………………………………………………………………………….23

WILEY-VCH

Materials

The following chemicals were used without any modifications :fumed silica (Cab-O-Sil M7D, cabot), *N,N,N*-Trimethyl-(–)-cis-myrtanylammonium hydroxide (TMMAOH, Mitsubishi chemical, 39%) as an organic structure-directing agent, sodium hydroxide aqueous solution (8 M NaOH aq., FUJIFILM Wako Pure Chemical), boric acid (FUJIFILM Wako Pure Chemical), aluminum sulfate (FUJIFILM Wako Pure Chemical, 85%), Beta zeolite (Si/Al=12.5, CP814E, Zeolyst), MFI-type zeolite (Si/Al=11.5, HSZ-820-NHA, Tosoh), FAU-type zeolite (Si/Al=15, CBV720, Zeolyst) and CIT-1, [Al, B]-CON-type zeolite as a seed crystal (Si/Al = 270, Mitsubishi Chemical).

Preparation of the CON-type zeolite

Synthesis of CON-400

The CON-type aluminosilicate zeolites [Al,B]-CON were hydrothermally synthesized from the aqueous gel containing a *N,N,N*-Trimethyl-(–)-cis-myrtanylammonium hydroxide, NaOH, boric acid, aluminum sulfate, fumed silica and 2 wt % of CIT-1, [Al, B]-CON-type zeolite (Si/Al = 270) as a seed crystal. The molar composition of the resulting mother gel was 1 SiO_2 : 0.1 H_3BO_3 : 0.0013 $Al_2(SO_4)_3$: 0.1 NaOH : 0.2 TMMAOH : 30 H_2O. The prepared gel was crystallized at 170 °C for 7 days according to the previous report [1, 2].

Synthesis of CON-Beta-40 & CON-MFI-40

N,N,N-Trimethyl-(–)-cis-myrtanylammonium hydroxide (TMMAOH) was used as received from Mitsubishi Chemical Co. Ltd., Japan, i.e., as an aqueous solution (30.9 wt%). To the 5.52 g (8 mmol) of TMMAOH aq., 0.64 g (4 mmol) of sodium hydroxide aqueous solution (NaOH aq., 8 M, FUJIFILM Wako Pure Chemical Co. Ltd., Japan) and deionized H_2O (17.3 g) were added and stirred. Then, boric acid (0.25 mg, 4 mmol, FUJIFILM Wako Pure Chemical Co. Ltd., Japan) was added. After 1 h of stirring, the white suspension obtained was treated with fumed silica (1.60 g, 27 mmol, Cab-O-Sil M7D, Cabot Corporation, USA), before stirring was continued for another hour. After Beta zeolite (0.80 g, 13 mmol, Si/Al=12.5, CP814E, Zeolyst) or MFI-type zeolite (0.80 g, 13 mmol, Si/Al=11.5, HSZ-820NHA, Tosoh) was added as a starting zeolite. Then, stirring was continued for another hour. The molar composition of the resulting mother gel was 1 SiO_2 : 0.1 H_3BO_3 : 0.013 Al_2O_3 : 0.1 NaOH : 0.2 TMMAOH : 30 H_2O. The prepared gel was crystallized at 170 °C for 7 days under tumbling conditions (40 rpm).

Synthesis of CON-P-30

The CON-type borosilicate zeolite [B]-CON were hydrothermally synthesized from the aqueous gel containing a *N,N,N*-Trimethyl-(–)-cis-myrtanylammonium hydroxide, NaOH, boric acid, aluminum sulfate, fumed silica and 2 wt % of CIT-1, [Al, B]-CON-type zeolite (Si/Al = 270) as a seed crystal. The molar composition of the resulting mother gel was 1 SiO_2 : 0.1 H_3BO_3 : 0.1 NaOH : 0.2 TMMAOH : 30 H_2O. The prepared gel was crystallized at 170 °C for 7 days according to the previous report [1, 3]. The deboronation of [B]-CON (Si/B = 30) was conducted by the acid treatment using 0.05 M HCl for 24 h at 130 °C. The deboronated sample (Si/B >300) was treated in the aqueous solution containing aluminum nitrate for 12 h at 130 °C with the molar ratios of 1 SiO_2 :0.64 Al $(NO_3)_3$:167 H_2O.

Synthesis of CON-Beta+MFI-20

N,N,N-Trimethyl-(–)-cis-myrtanylammonium hydroxide (TMMAOH) was used as received from Mitsubishi Chemical Co. Ltd., Japan, i.e., as an aqueous solution (30.9 wt%). To the 5.52 g (8 mmol) of TMMAOH aq., 0.64 g (4 mmol) of sodium hydroxide aqueous solution (NaOH aq., 8 M, FUJIFILM Wako Pure Chemical Co. Ltd., Japan) and deionized H_2O (17.3 g) were added and stirred. Then, boric acid (0.25 mg, 4 mmol, FUJIFILM Wako Pure Chemical Co. Ltd., Japan) was added. After 1 h of stirring, the white suspension obtained was treated with fumed silica (0.9 g, 15 mmol, Cab-O-Sil M7D, Cabot Corporation, USA), before stirring was continued for another hour. After Beta zeolite (0.80 g, 13 mmol, Si/Al=12.5, CP814E, Zeolyst) and MFI-type zeolite (0.80 g, 13 mmol, Si/Al=11.5, HSZ-820NHA, Tosoh) was added as a starting zeolite. Then, stirring was continued for another hour. The molar composition of the resulting mother gel was 1 SiO_2 : 0.1 H_3BO_3 : 0.025 Al_2O_3 : 0.1 NaOH : 0.2 TMMAOH : 30 H_2O. The prepared gel was crystallized at 200 °C for 7 days under tumbling conditions (40 rpm).

Synthesis of CON-Beta: MFI=9:1

WILEY-VCH

N,N,N-Trimethyl-(–)-cis-myrtanylammonium hydroxide (TMMAOH) was used as received from Mitsubishi Chemical Co. Ltd., Japan, i.e., as an aqueous solution (30.9 wt%). To the 5.52 g (8 mmol) of TMMAOH aq., 0.64 g (4 mmol) of sodium hydroxide aqueous solution (NaOH aq., 8 M, FUJIFILM Wako Pure Chemical Co. Ltd., Japan) and deionized H_2O (17.3 g) were added and stirred. Then, boric acid (0.25 mg, 4 mmol, FUJIFILM Wako Pure Chemical Co. Ltd., Japan) was added. After 1 h of stirring, the white suspension obtained was treated with fumed silica (0.9 g, 15 mmol, Cab-O-Sil M7D, Cabot Corporation, USA), before stirring was continued for another hour. After Beta zeolite (0.72 g, 11.7 mmol, Si/Al=12.5, CP814E, Zeolyst) and MFI-type zeolite (0.08 g, 1.3 mmol, Si/Al=11.5, HSZ-820NHA, Tosoh) was added as a starting zeolite. Then, stirring was continued for another hour. The molar composition of the resulting mother gel was 1 SiO_2 : 0.1 H_3BO_3 : 0.025 Al_2O_3 : 0.1 NaOH : 0.2 TMMAOH : 30 H_2O.The prepared gel was crystallized at 200 °C for 7 days under tumbling conditions (40 rpm).

Synthesis of CON-Beta: MFI=1:9

N,N,N-Trimethyl-(–)-cis-myrtanylammonium hydroxide (TMMAOH) was used as received from Mitsubishi Chemical Co. Ltd., Japan, i.e., as an aqueous solution (30.9 wt%). To the 5.52 g (8 mmol) of TMMAOH aq., 0.64 g (4 mmol) of sodium hydroxide aqueous solution (NaOH aq., 8 M, FUJIFILM Wako Pure Chemical Co. Ltd., Japan) and deionized H_2O (17.3 g) were added and stirred. Then, boric acid (0.25 mg, 4 mmol, FUJIFILM Wako Pure Chemical Co. Ltd., Japan) was added. After 1 h of stirring, the white suspension obtained was treated with fumed silica (0.9 g, 15 mmol, Cab-O-Sil M7D, Cabot Corporation, USA), before stirring was continued for another hour. After Beta zeolite (0.08 g, 1.3 mmol, Si/Al=12.5, CP814E, Zeolyst) and MFI-type zeolite (0.72 g, 11.7 mmol, Si/Al=11.5, HSZ-820NHA, Tosoh) was added as a starting zeolite. Then, stirring was continued for another hour. The molar composition of the resulting mother gel was 1 SiO_2 : 0.1 H_3BO_3 : 0.025 Al_2O_3 : 0.1 NaOH : 0.2 TMMAOH : 30 H_2O.The prepared gel was crystallized at 200 °C for 7 days under tumbling conditions (40 rpm).

WILEY-VCH

Characterization

The crystallinity of the structures of the synthesized samples was confirmed based on their X-ray diffraction (XRD) patterns (Rigaku Ultima III diffractometer, Cu Ka radiation, 40 kV, 40 mA). Field emission scanning electron microscopy (FE-SEM) images of the samples were obtained on Hitachi S-5200 or SU9000 microscopes operated at 1 kV. Elemental analysis of the samples (Si/Al and Si/B molar ratios) was performed using an inductively coupled plasma-atomic emission spectroscopy (ICP-AES, Shimadzu ICPE-9000). The Brunauer–Emmett–Teller (BET) specific surface areas and micropore volumes were calculated from the adsorption branch data of the nitrogen adsorption–desorption measurements obtained from a BELSORP-MAX (MicrotracBEL). Samples were treated at 623 K for 3 h prior to the measurements. Temperature-programmed NH_3 desorption (NH_3-TPD) profiles were recorded on a BELCAT-A (MicrotracBEL). A thermal conductivity detector (TCD) was used to monitor the desorbed NH_3. The amount of acidic sites was determined using the area of the so-called "*h*-peak" in the profiles. High-resolution ^{27}Al MAS NMR and ^{27}Al 3Q MQMAS NMR spectra were obtained on a JEOL ECA-600 spectrometer (14.1 T) equipped with an additional 1 kW power amplifier using a ZrO_2 rotor (4 mm in size) under ambient conditions. For the ^{27}Al 3Q MQMAS NMR spectra, the 3Q excitation pulse and the 3Q-1Q conversion pulse were 5.5 and 2.1 μs, respectively, while the z-filter was set to 0.2 ms. The relaxation delay was 10 ms. The ^{27}Al NMR shifts were referenced to $AlNH_4(SO_4)_2 \cdot 12H_2O$ at −0.54 ppm. The samples were spun at 15 kHz using a 4 mm ZrO_2 rotor.

Cracking of hexane

Hexane cracking reactions were conducted within a fixed-bed quartz reactor employing a continuous flow system under atmospheric pressure. Before the reaction, catalysts were crushed and sieved, and 20 mg of pelletized catalysts (20–40 mesh) was activated in flowing Ar at 823 K for 1 h and then cooled to the desired reaction temperature (873 K). The flow rate of carrier gas was set at 6.2 mL/min, the flow rate of n-hexane was set at 2.1 μL/min. The products were analyzed by utilizing an online gas chromatograph (Shimadzu GC-14B) featuring a flame ionization detector (FID).

Cracking of 1,3,5-Triisopropylbenzene

To assess the alteration of acid sites presented on the external surface, the cracking of 1,3,5-triisopropylbenzene (TIPB) (Sigma-Aldrich, 99%) was carried out. These reactions were conducted within a fixed-bed quartz reactor employing a continuous flow system under atmospheric pressure. Before the reaction, catalysts were crushed and sieved, and 20 mg of pelletized catalysts (20–40 mesh) was activated in flowing Ar at 823 K for 1 h and then cooled to the desired reaction temperature (673 K). The flow rate of carrier gas was set at 10 mL/min, the flow rate of TIPB was set at 10 μL/min, and the reaction time was 10 min. The products were analyzed by utilizing an online gas chromatograph (Shimadzu 14B) featuring a flame ionization detector (FID). Chromatographic separation was achieved by using a 30 m DB-1 capillary column.

WILEY-VCH

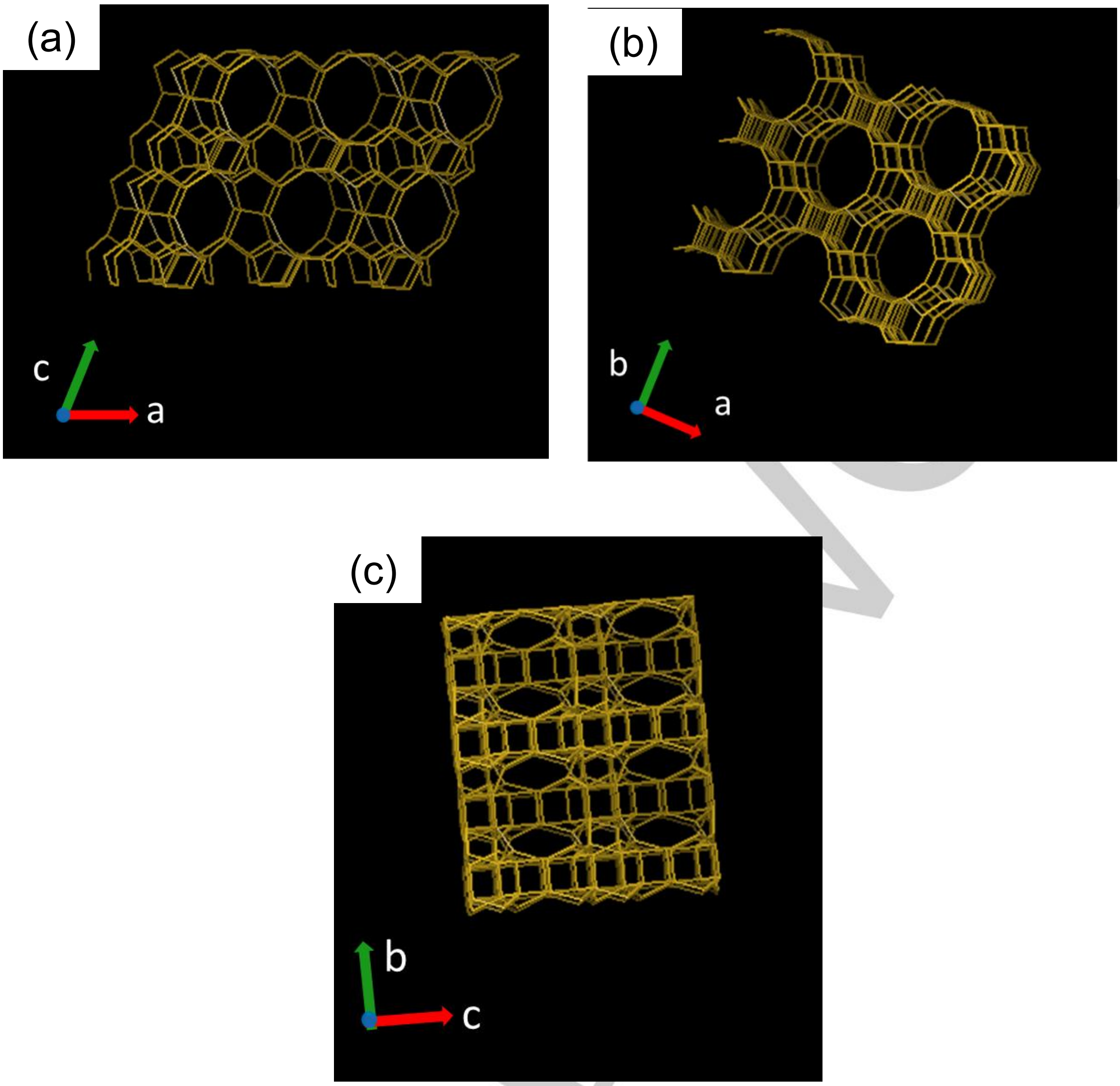

Figure S1. Framework structures of CON-phase; (a) viewed along b-axis, (b) viewed along c-axis, and (c) viewed along a-axis.

WILEY

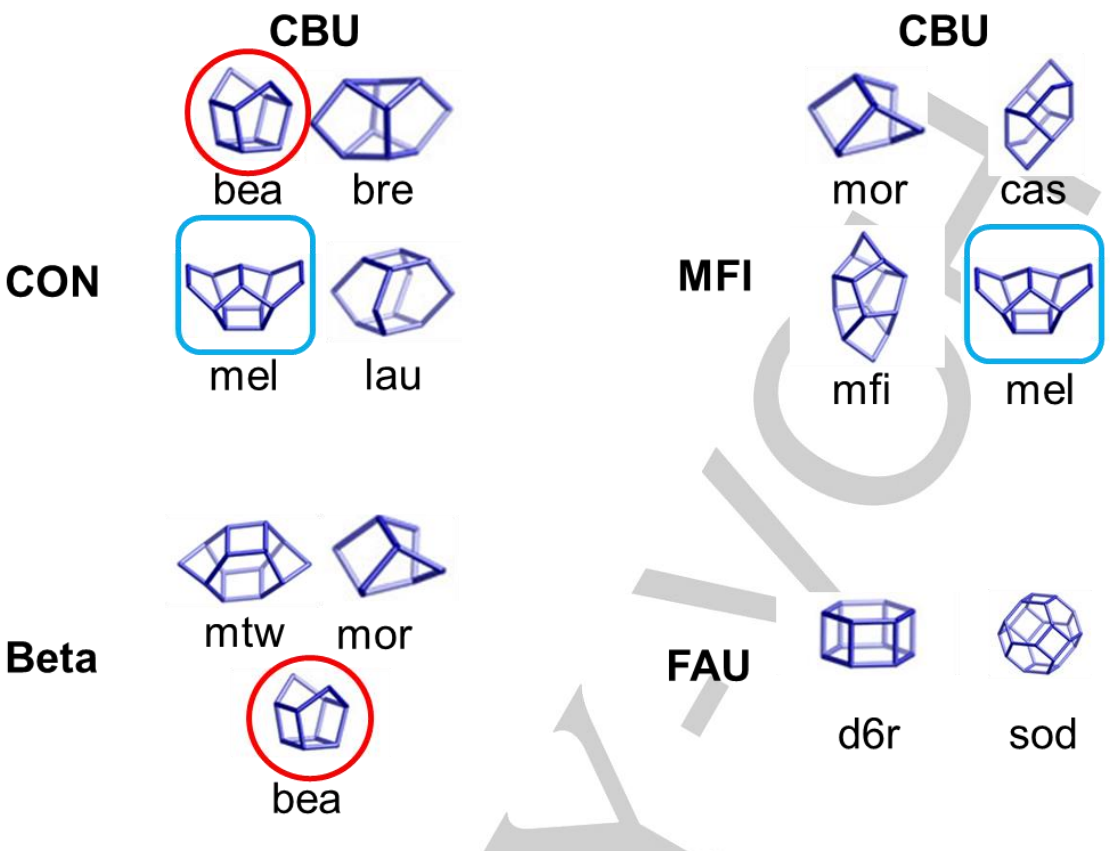

Figure S2. Composite building units (CBU) of CON-type, Beta, MFI-type and FAU-type zeolites; Red circle, common CBU in CON-type and Beta zeolite; blue square, common CBU in CON-type and MFI-type zeolite.

WILEY-VCH

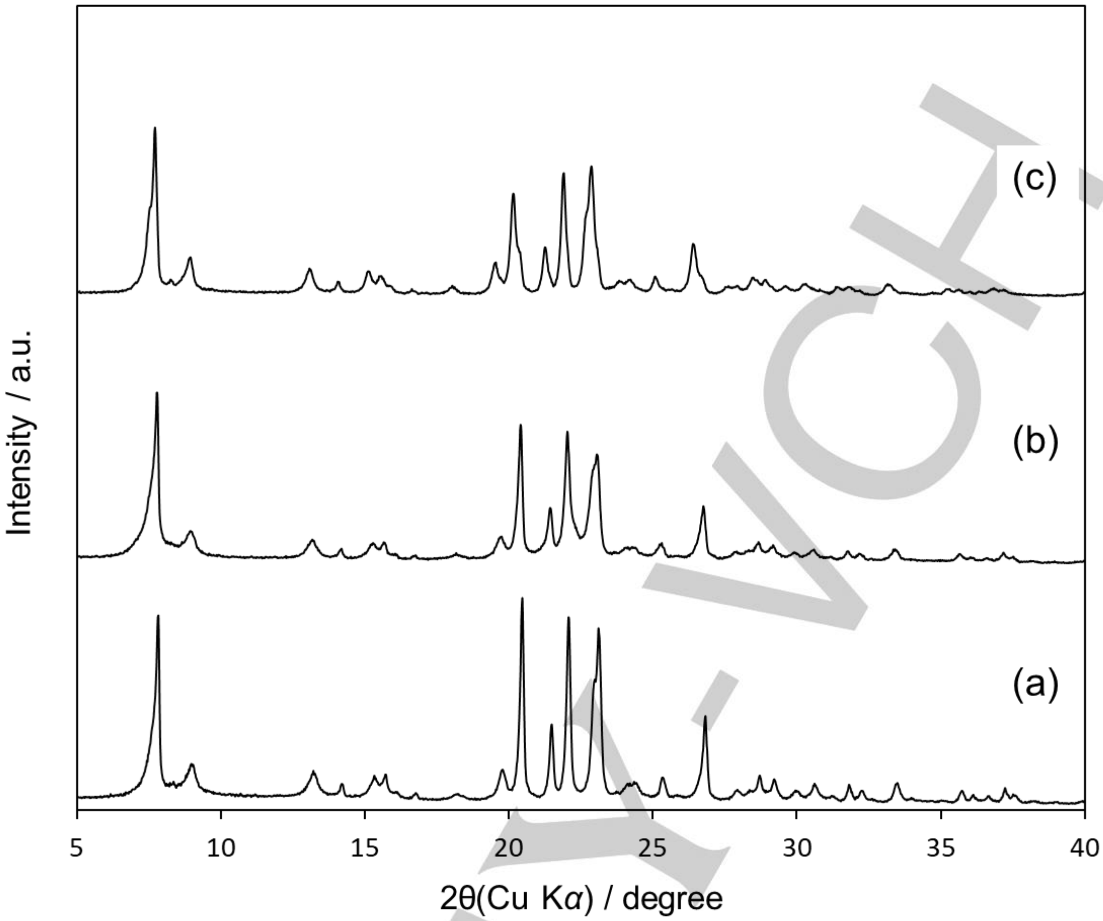

Figure S3. XRD patterns of CON-type zeolites; (a) CON-400, (b) CON-Beta-40-2days and (c) CON-Beta-40-7days.

WILEY-VCH

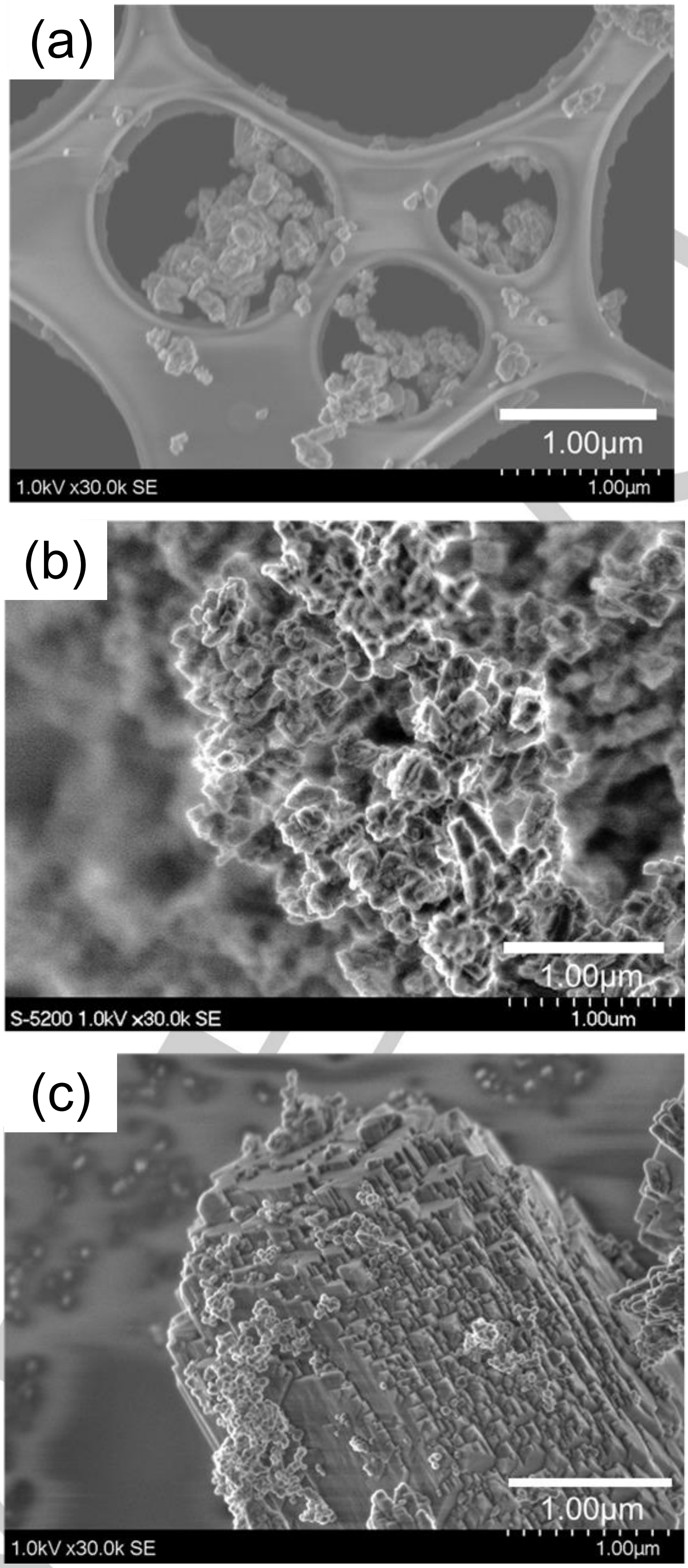

Figure S4. SEM images of CON-type zeolites; (a) CON-400, (b) CON-Beta-40, and (c) CON-MFI-40.

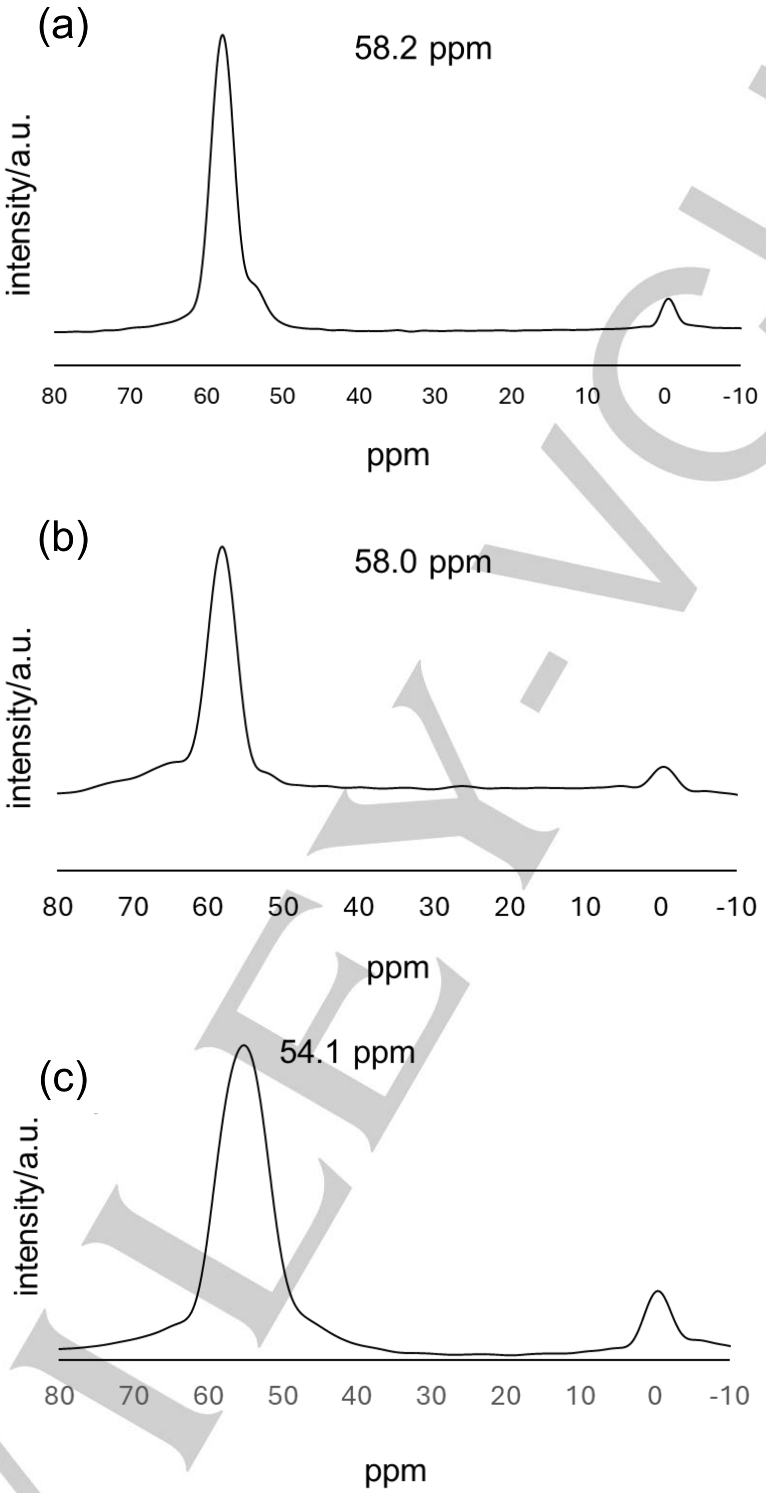

Figure S5. ^{27}Al MAS NMR spectra; (a) CON-400, (b) CON-Beta-40, and (c) CON-MFI-40.

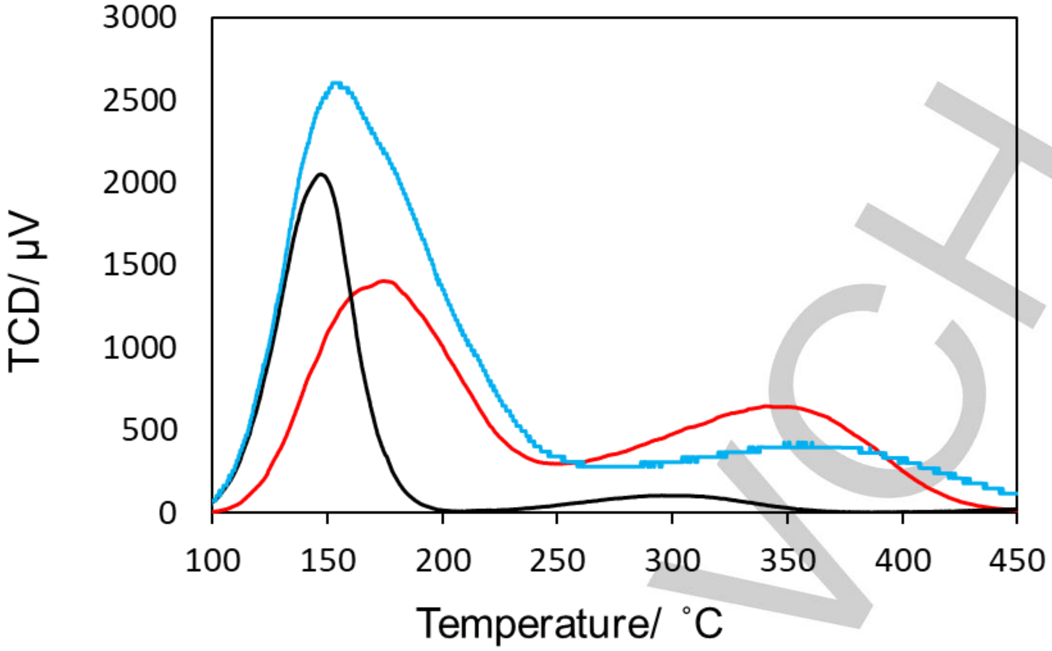

Figure S6. NH_3-TPD profiles; black line, CON-400; red line, CON-Beta-40; and blue line, CON-MFI-40.

WILEY-VCH

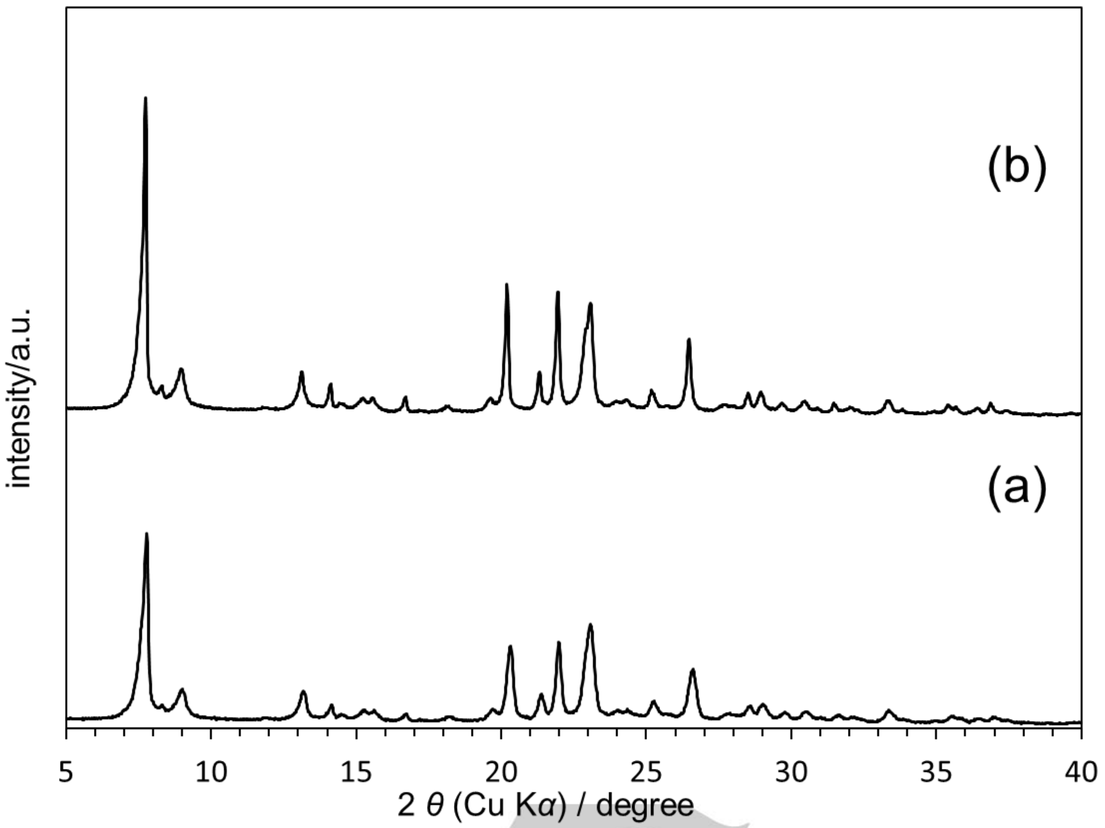

Figure S7. XRD patterns of CON-type zeolites; (a) CON-Beta-40 and (b) CON-P-30.

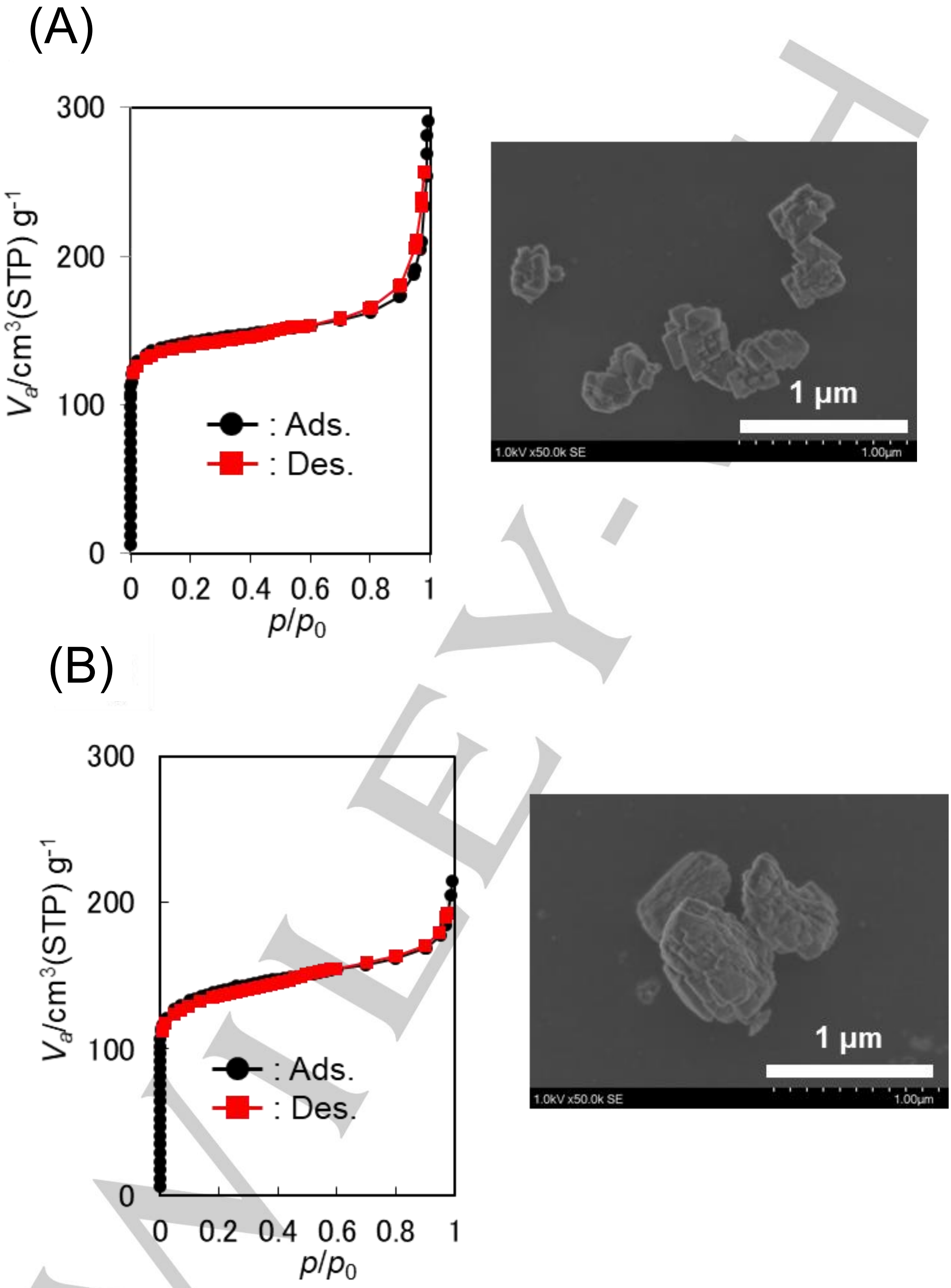

Figure S8. N_2 adsorption & desorption isotherm and SEM images of CON-type zeolites (A) CON-Beta-40 and (B) CON-P-30.

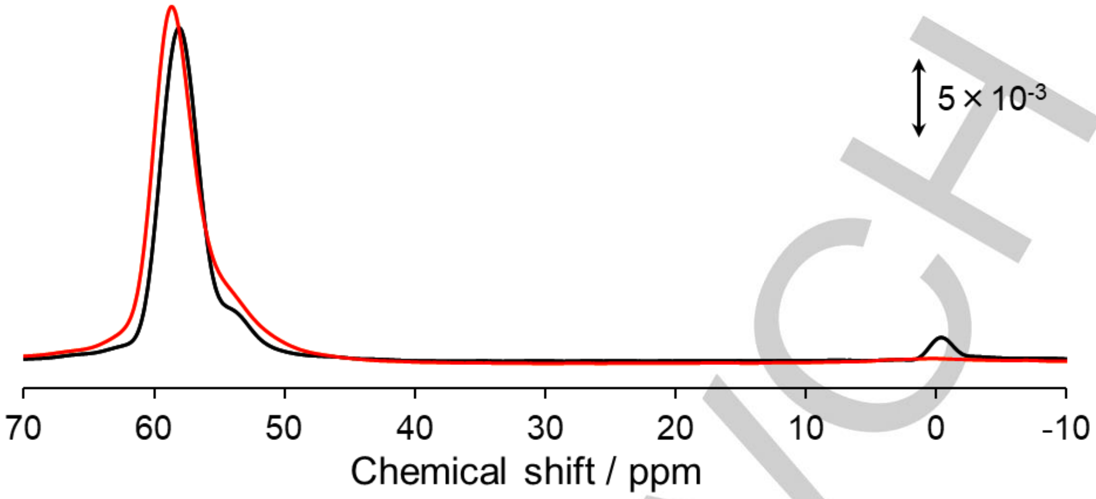

Figure S9. ^{27}Al MAS NMR spectra; black line, CON-Beta-40 (–) and red line, CON-P-30 (–).

WILEY-VCH

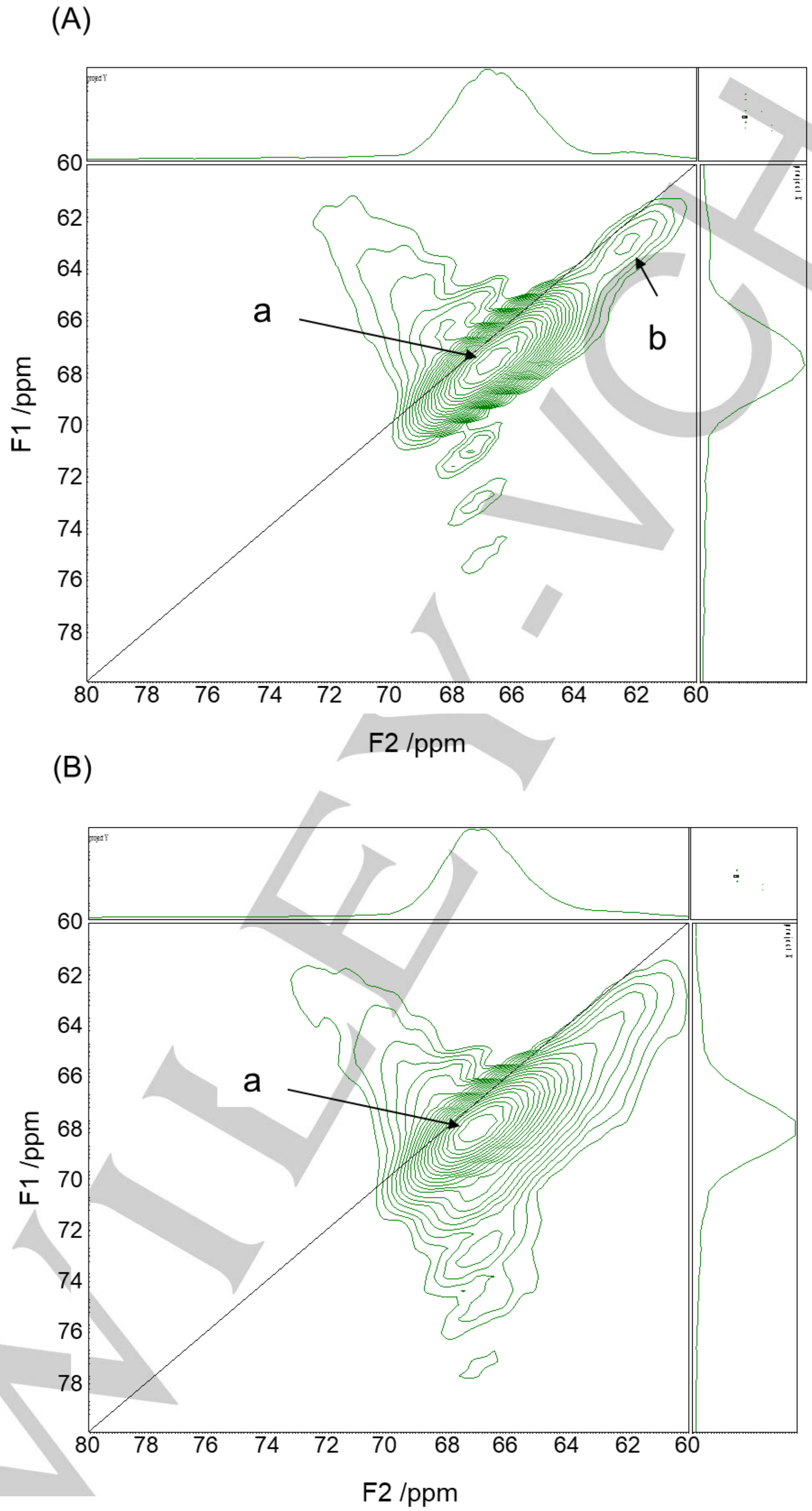

Figure S10. ^{27}Al 3QMQMAS NMR spectra of (A) CON-Beta-40 and (B) CON-P-30.

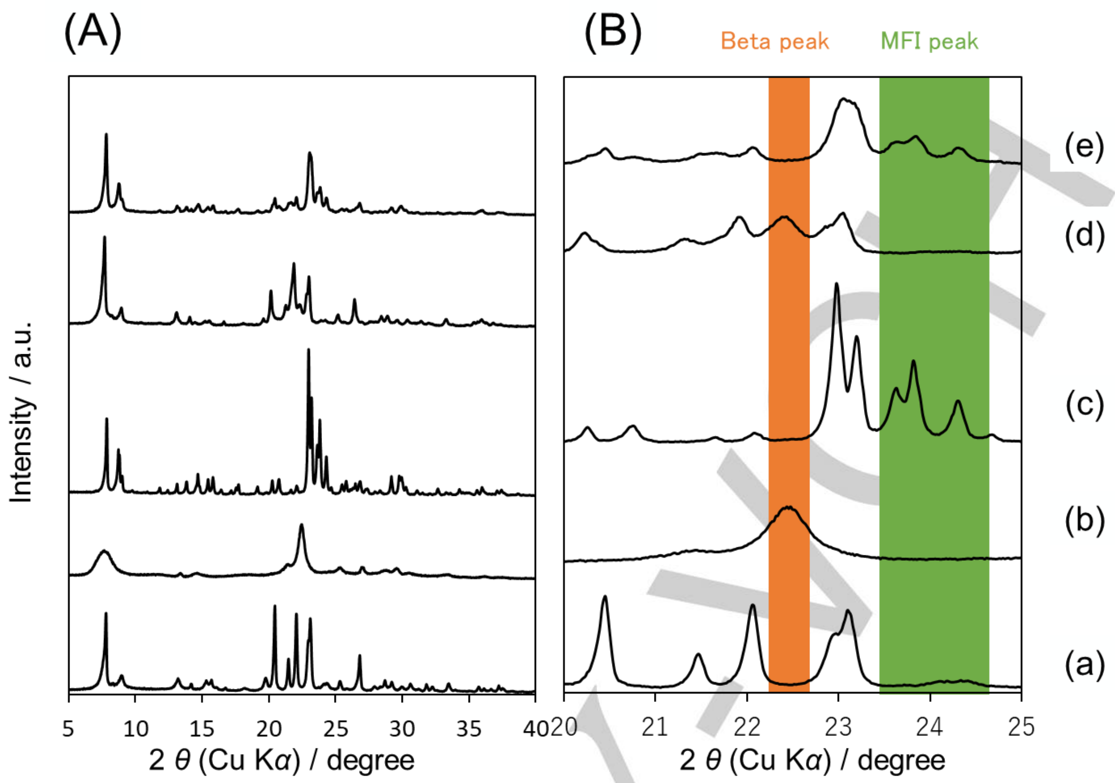

Figure S11. XRD patterns of CON-type zeolites and reference zeolites around the range of (A) 2θ = 5–40 and (B) 2θ = 20–25 deg.; (a) CON-400, (b) Si/Al=12.5 Beta, (c) Si/Al=11.5 MFI, (d) CON-Beta-20, and (e) CON-MFI-20.
(d) was synthesized by using Beta-zeolite for synthesis CON-type zeolite with Si/Al ratio of 20, (e) was synthesized by using Beta-zeolite for synthesis CON-type zeolite with Si/Al ratio of 20.

WILEY-VCH

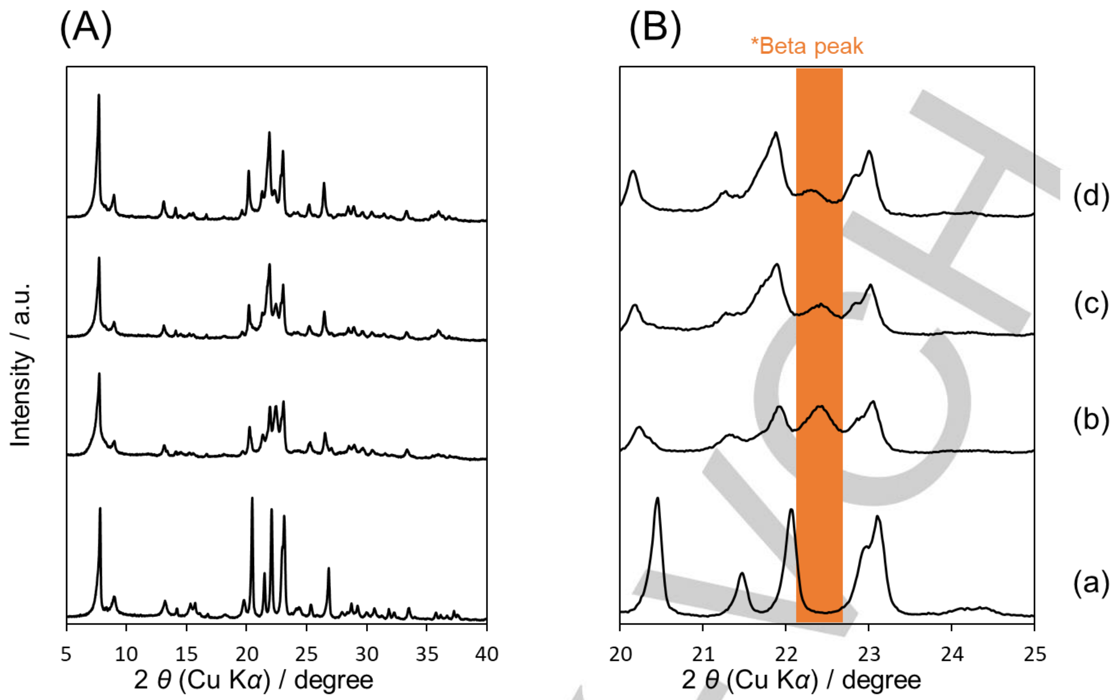

Figure S12. XRD patterns of CON-type zeolites around the range of (A) 2θ = 5–40 and (B) 2θ = 20–25 deg.; (a) CON-400, (b) CON-Beta-20-170 °C -7d, (c) CON-Beta-20-200 °C -7d, and (d) CON-Beta-20-200 °C -10d.
(b) was synthesized by using Beta-zeolite for synthesis CON-type zeolite with Si/Al ratio of 20 at 170 °C 7 days, (c) was synthesized by using Beta-zeolite for synthesis CON-type zeolite with Si/Al ratio of 20 at 200 °C 7 days, (d) was synthesized by using Beta-zeolite for synthesis CON-type zeolite with Si/Al ratio of 20 at 200 °C 10 days.

WILEY-VCH

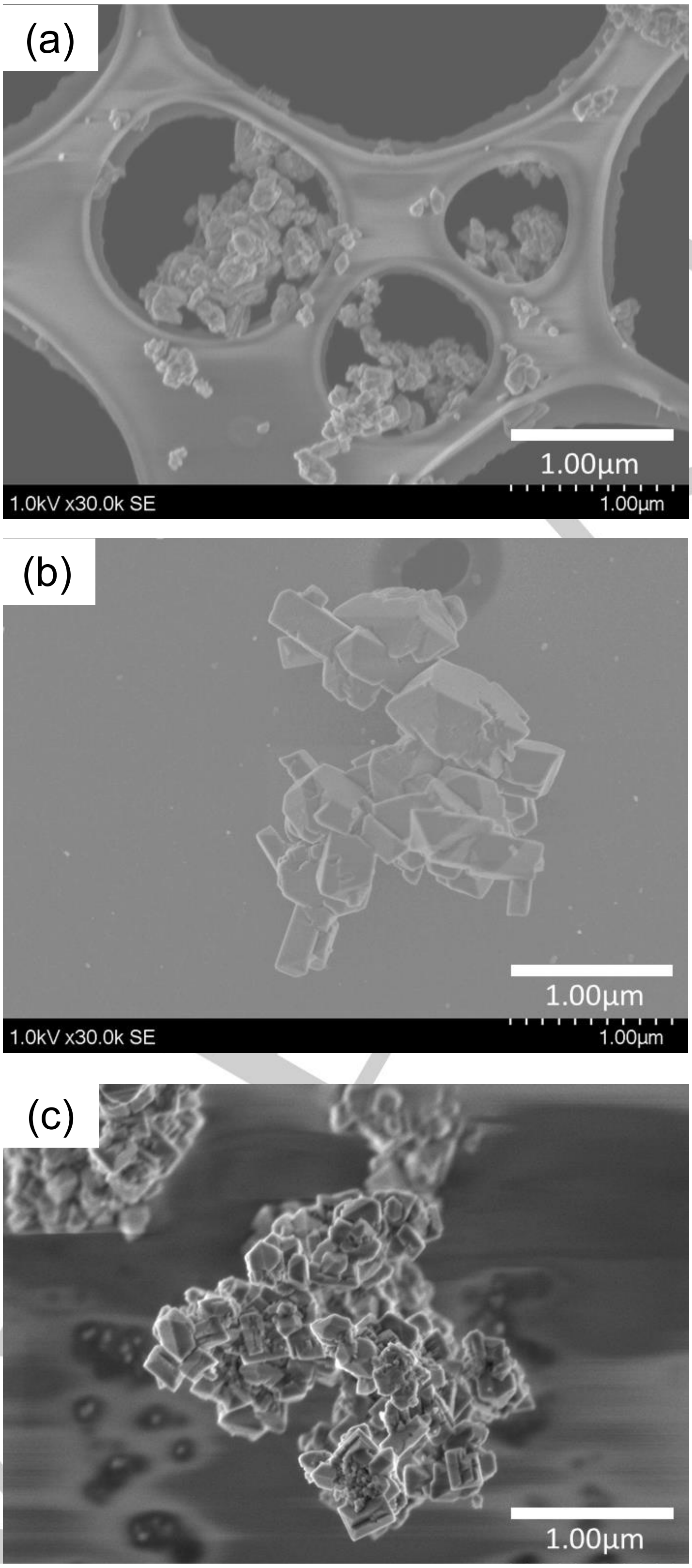

Figure S13. SEM images of CON-type zeolites; (a) CON-400 and (b) CON-Beta+MFI-20, and (c) CON-Beta+MFI-20-170 °C.

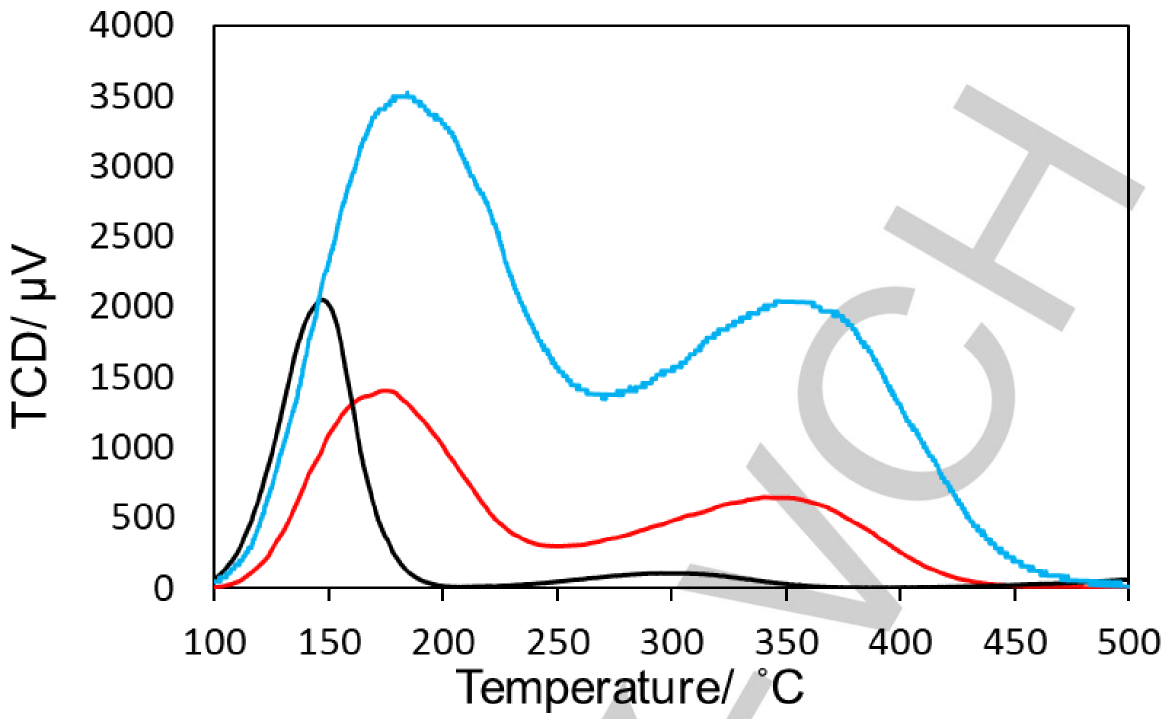

Figure S14. NH_3-TPD profiles; black line: CON-400, red line: CON-Beta-40, and blue line: CON-Beta+MFI-20.

WILEY-VCH

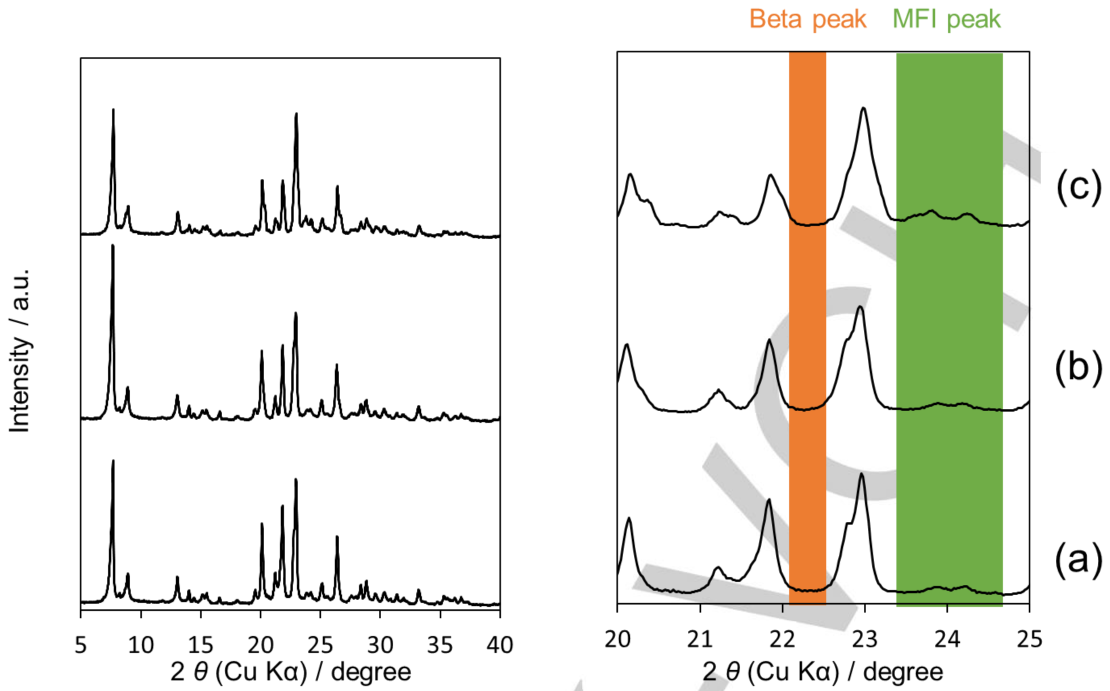

Figure S15. XRD patterns of CON-type zeolites around the range of (A) 2θ = 5–40 and (B) 2θ = 20–25 deg.; (a) CON-Beta: MFI=9:1, (b) CON-Beta: MFI=1:1, and (c) CON-Beta: MFI=1:9.

WILEY-VCH

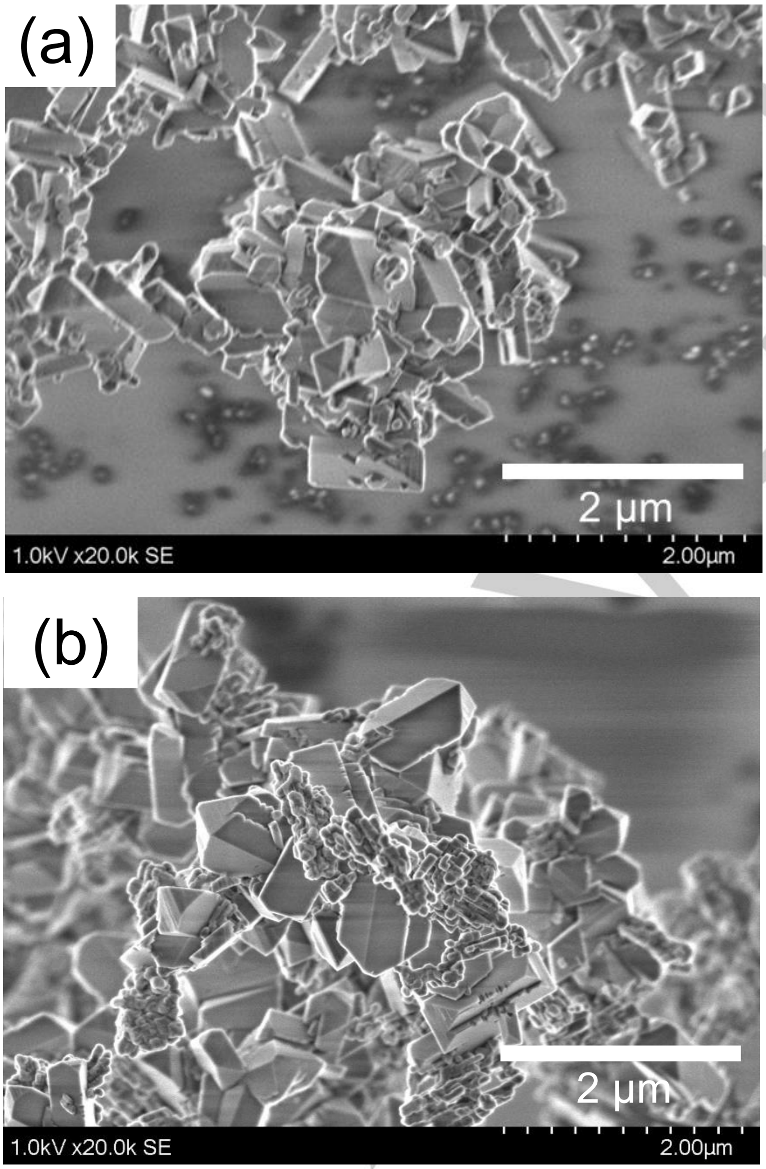

Figure S16. SEM images of CON-type zeolites; (a) CON-Beta: MFI=9:1 and (b) CON-Beta: MFI=1:9.

WILEY-VCH

Table S1. Physicochemical properties of the direct and post synthesis CON-type zeolites.

CON-	Si/Al[a]	Si/B[a]	A_{acid}[b] /mmol (g-cat)$^{-1}$	S_{BET}[c] /m2g^{-1}	V_{micro}[c] /cm^{3}g^{-1}	S_{ext}[c] /m^{2}g^{-1}
Beta-40	41	51	0.270	670	0.24	46
P-30	28	>1000	0.342	520	0.22	29

[a]ICP-AES, [b]NH_3-TPD, and [c]N_2 adsorption-desorption measurement

WILEY-VCH

Table S2. Physicochemical properties of the CON-type zeolites by blending method.

CON-	Si/Al[a]	Si/B[a]	A_{acid}[b] /mmol (g-cat)$^{-1}$	S_{BET}[c] /m2g^{-1}	V_{micro}[c] /cm^{3}g^{-1}	S_{ext}[c] /m^{2}g^{-1}
Beta+MFI-20	19	101	0.560	698	0.26	16
Beta-40	41	51	0.270	670	0.24	46
400	431	31	0.018	633	0.24	43

[a]ICP-AES, [b]NH_3-TPD, and [c]N_2 adsorption-desorption

WILEY-VCH

Supplementary References

[1] M. Yoshioka, T. Yokoi, T. Tatsumi, *ACS Catalysis* **2015**, 5, 4268-4275.
[2] M. Sawada, T. Matsumoto, R. Osuga, S. Yasuda, S. Park, Y. Wang, J. N. Kondo, H. Onozuka, S. Tsutsuminai, T. Yokoi, *Industrial & Engineering Chemistry Research* **2022**, 61, 1733-1747.
[3] R. F. Lobo, Mark E. Davis, *Journal of the American Chemical Society* **1995**, 117, 3766-3779.

WILEY-VCH
